# Moving the needle: Employing deep reinforcement learning to push the boundaries of coarse-grained vaccine models

**DOI:** 10.3389/fimmu.2022.1029167

**Published:** 2022-11-03

**Authors:** Jonathan G. Faris, Daniel Orbidan, Charles Wells, Brenden K. Petersen, Kayla G. Sprenger

**Affiliations:** ^1^ Department of Chemical and Biological Engineering, University of Colorado Boulder, Boulder, CO, United States; ^2^ Department of Computer Science, Rice University, TX, Houston, United States; ^3^ Computational Engineering Division, Lawrence Livermore National Laboratory, Livermore, CA, United States

**Keywords:** deep reinforcement learning (Deep RL), agent-based modelling, multiscale (MS) modelling, affinity maturation, HIV - human immunodeficiency virus, vaccine design protocol, immunovirology

## Abstract

Highly mutable infectious disease pathogens (hm-IDPs) such as HIV and influenza evolve faster than the human immune system can contain them, allowing them to circumvent traditional vaccination approaches and causing over one million deaths annually. Agent-based models can be used to simulate the complex interactions that occur between immune cells and hm-IDP-like proteins (antigens) during affinity maturation—the process by which antibodies evolve. Compared to existing experimental approaches, agent-based models offer a safe, low-cost, and rapid route to study the immune response to vaccines spanning a wide range of design variables. However, the highly stochastic nature of affinity maturation and vast sequence space of hm-IDPs render brute force searches intractable for exploring all pertinent vaccine design variables and the subset of immunization protocols encompassed therein. To address this challenge, we employed deep reinforcement learning to drive a recently developed agent-based model of affinity maturation to focus sampling on immunization protocols with greater potential to improve the chosen metrics of protection, namely the broadly neutralizing antibody (bnAb) titers or fraction of bnAbs produced. Using this approach, we were able to coarse-grain a wide range of vaccine design variables and explore the *relevant* design space. Our work offers new testable insights into how vaccines should be formulated to maximize protective immune responses to hm-IDPs and how they can be minimally tailored to account for major sources of heterogeneity in human immune responses and various socioeconomic factors. Our results indicate that the first 3 to 5 immunizations, depending on the metric of protection, should be specially tailored to achieve a robust protective immune response, but that beyond this point further immunizations require only subtle changes in formulation to sustain a durable bnAb response.

## Introduction

Vaccination has saved more lives to date than any other medical procedure (1), and yet viruses capable of evading traditional vaccination schemes continue to emerge, often with devastating consequences. We have seen the rise of three global pandemics in the last 100 years (COVID-19, HIV/AIDS, and the 1918 Spanish Flu), which have cumulatively claimed 90 million lives (2–4). The highly mutable infectious disease pathogens (hm-IDPs) that cause these diseases present many technical challenges to traditional vaccination approaches, including the ability of hm-IDPs to rapidly mutate their surface proteins (antigens) to escape immune pressure (5). In turn, these technical challenges lead to broader societal challenges such as healthcare inequalities that arise in relation to the frequency with which new vaccines must be developed and distributed to keep pace with, and provide protection against, new variants (6). Consequently, the need for next-generation immunotherapies to combat existing and future hm-IDPs has never been greater.

Two schools of thought have since arisen to overcome the aforementioned challenges presented by hm-IDPs: universal vaccines and personalized medicine. Progress towards universal vaccines continues to advance every year, with increasingly informed design rules on how to elicit antibodies that target conserved aspects of viral machinery (7–14). Much of this newfound knowledge has come from studying crystal structures of broadly neutralizing antibodies (bnAbs)—which neutralize diverse viral strains by targeting conserved regions on hm-IDP surface proteins—and attempting to reconstruct the evolutionary pathways of these bnAbs (15–26). In the past few decades, bnAbs have been isolated from individuals chronically infected with hm-IDPs that, for instance, target the conserved stalk and head regions on influenza’s hemagglutinin spike protein (27, 28) or the conserved loops of HIV’s Env spike protein (29–32). While bnAbs serve as ideal targets for universal vaccines, challenges remain around how to robustly elicit them *via* vaccination.

Questions about universal vaccines also exist in terms of how efficacious they would be for diverse subpopulations. A single universal vaccine may not be able to capture major sources of heterogeneity in human immune responses to hm-IDPs (33–35). For example, compared to adults, infants and children typically harbor an abundance of naïve B cells (36) and experience higher viral replication rates and viral loads upon infection with HIV during birth (37–40), all of which could influence the quantity and quality of antibodies produced in response to a vaccine. Further, optimal metrics of protection against hm-IDPs are often unclear. For instance, bnAb titers are among the most common and useful clinical measures of protection. However, multiple studies have now shown a quality-quantity tradeoff exists whereby the affinity maturation (AM) process by which antibodies evolve produces either low titers of high-breadth antibodies or high titers of low-breadth antibodies (13, 24, 41). It is unclear which outcome may offer better protection for different individuals and/or hm-IDPs (e.g., maximizing the *fraction* of bnAbs, or maximizing the *titer* of bnAbs). Additionally, for a successful vaccination strategy, the number of immunizations required must be considered and perhaps tailored for areas of both high and low vaccine adherence (42, 43).

On the opposite end of the spectrum from universal vaccines, precision medicine approaches seek to derive personalized care and treatment plans based on an individual’s genetics and disease progression. These approaches are being increasingly explored for treating a variety of conditions such as cancer (44, 45), sepsis (46, 47), and HIV/AIDS (48, 49). However, the price tag for these personalized therapies can be shocking, with costs to an individual of more than $10,000 a month based on the results of genomic sequencing, which itself can be very costly (50). Thus, whereas universal vaccines may not offer enough flexibility and tractability to broadly protect a large fraction of the human population, personalized medicine approaches may be overly-tailored, rendering them economically unviable on a global scale. We propose a solution that lies between these two extremes, which is to develop a minimal set of vaccines designed to elicit bnAbs (and hence that are still “universal”) that can collectively account for a broad range of differences in human immune responses and socioeconomic factors.

Developing a universal vaccine against a specific hm-IDP requires large-scale screening of vaccine-candidate antigens and extensive laboratory resources to determine the optimal vaccine design parameters. To circumvent these challenges, computational agent-based models can be employed to simulate the immune response upon exposure to hm-IDP-like antigens. These models are capable of recapitulating the complex immune population dynamics that take place during AM, which consists of alternating periods of somatic hypermutation (SHM) and selection for high-affinity B cell clones (51). Briefly, agent-based models use mathematical, empirically informed equations to describe how individuals, or *agents*, interact with each other and with their *environment*. In this work, B cells (the agents) compete with one another as AM (the environment) progresses in response to a series of vaccine-candidate antigens. The power in these models lies in their ability to capture highly stochastic processes, such as SHM, as the agents navigate the parameter space (e.g., total number of vaccine immunizations and number, concentration, and sequences of the administered antigens). For instance, a recent model of AM from Molari et al. showed the effects Ag concentration has on the speed by which BCRs mature (52). Each of these parameters has been shown to play an important role in modulating bnAb responses by affecting the level of *frustration* (see Methods) imposed on evolving antibodies (11–13, 24, 53–55), increases in which promote antibody targeting towards conserved antigenic residues but also promote B cell death.

**Figure 1:**
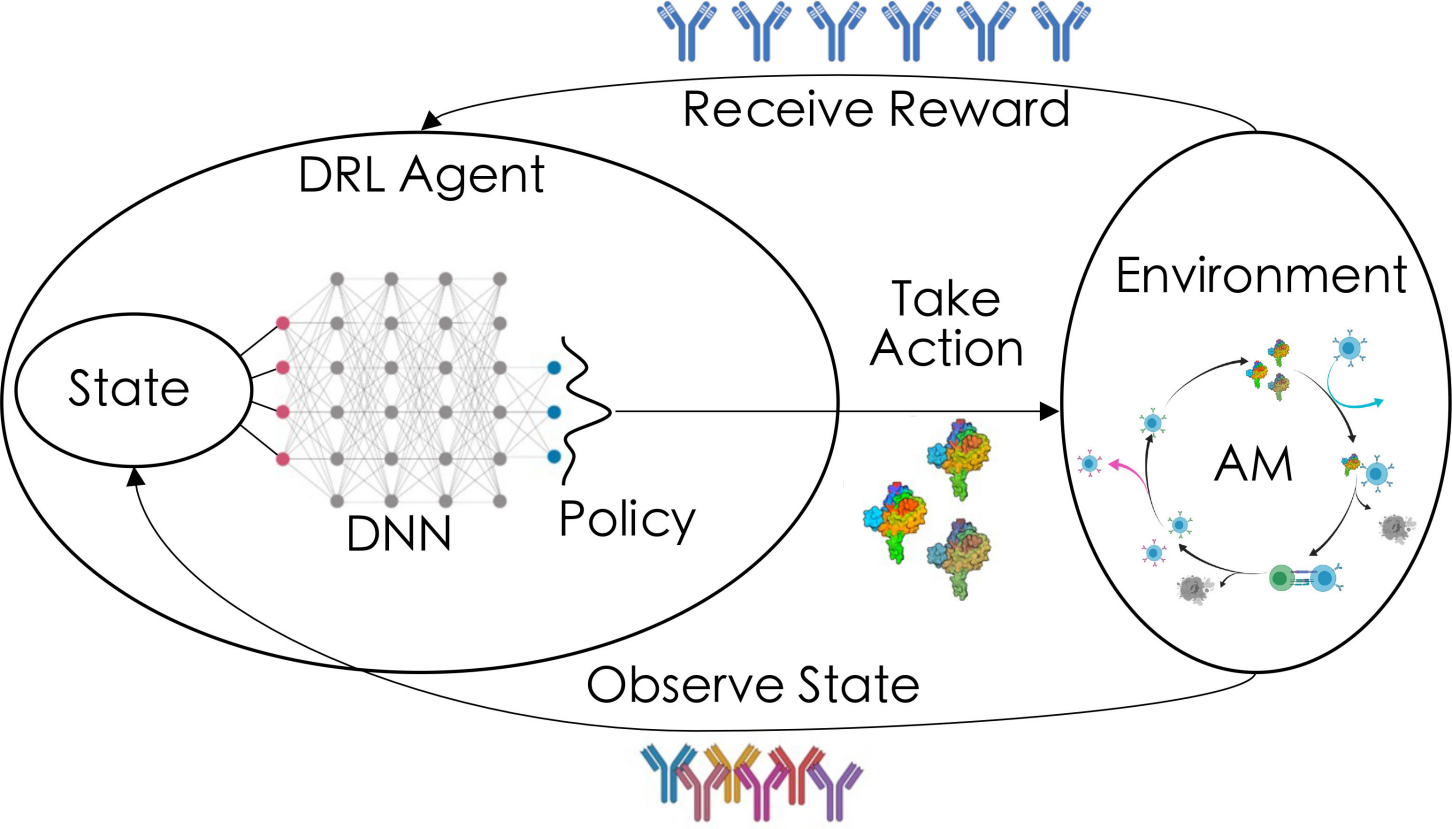
Overview of how deep reinforcement learning (DRL) is coupled to the agent-based model of affinity maturation (AM) in the current work. The *DRL agent* chooses an *action* (here, the level of vaccine-imposed frustration, modulated by changing antigen sequence and/or concentration) that is fed into the *environment* (here, the AM process), after which the agent observes the *state* of the system (here, properties of the vaccine-induced memory B cell receptor/antibody population). The agent then receives a *reward* based on how well the observed state meets a user-defined reward metric (here, the quality and quantity of the resulting plasma B cell receptor/antibody population). Over time, the agent learns a robust mapping between the states and actions, leading to an optimal *policy* (here, a vaccination/temporal frustration protocol) for maximizing the chosen reward.

Pushing beyond identifying the factors that *can* modulate bnAb responses, understanding *how* to rationally tune such parameters to maximize bnAb responses requires efficient exploration of this multidimensional parameter space. To this end, we have herein integrated a previously-published coarse-grained model of AM (13) with an established deep reinforcement learning (DRL) algorithm (**Figure 1**) (47, 56, 57). Briefly, when employing DRL, external *actions* are chosen throughout the simulation based on the observed *state* of the system, with the aim of guiding the agents along the path that maximizes a chosen *reward* function. After performing many simulations, DRL is able to learn a robust mapping between states and actions, and ultimately arrives at a *policy* (here, an optimal vaccination protocol) to maximize the overall reward. The approach taken here has been shown to provide novel solutions in the face of wide-ranging and complex problems such as optimization of DNA transcription factors (56), antimicrobial peptides (56), traffic signal controlling (57), and sepsis treatment (47). To the best of our knowledge, this work represents the first use of DRL to control a model of the adaptive immune response for designing vaccines against hm-IDPs.

In this work, the actions chosen by the DRL agent prescribe the level of *frustration*, defined by us in past work (13) to be a metric that quantitatively describes the combined effects of multiple vaccine design variables, on bnAb development. We explore how the policies chosen by DRL (e.g., optimal immunization protocols or temporal frustration profiles) change for different reward functions (metrics of protection), namely the total bnAb titers produced or fraction of bnAbs produced out of the total antibody titers. We also explore how the policies change as we vary the number of sequential immunizations in the vaccine protocol. The large policy differences we observe in some cases emphasize the need to develop a set of *minimally-tailored* universal vaccines against a given hm-IDP, which can account for important variabilities in immunoresponses based on differences in both immune and socioeconomic factors.

## Methods

### Affinity maturation model

#### Overview

**Figure 2:**
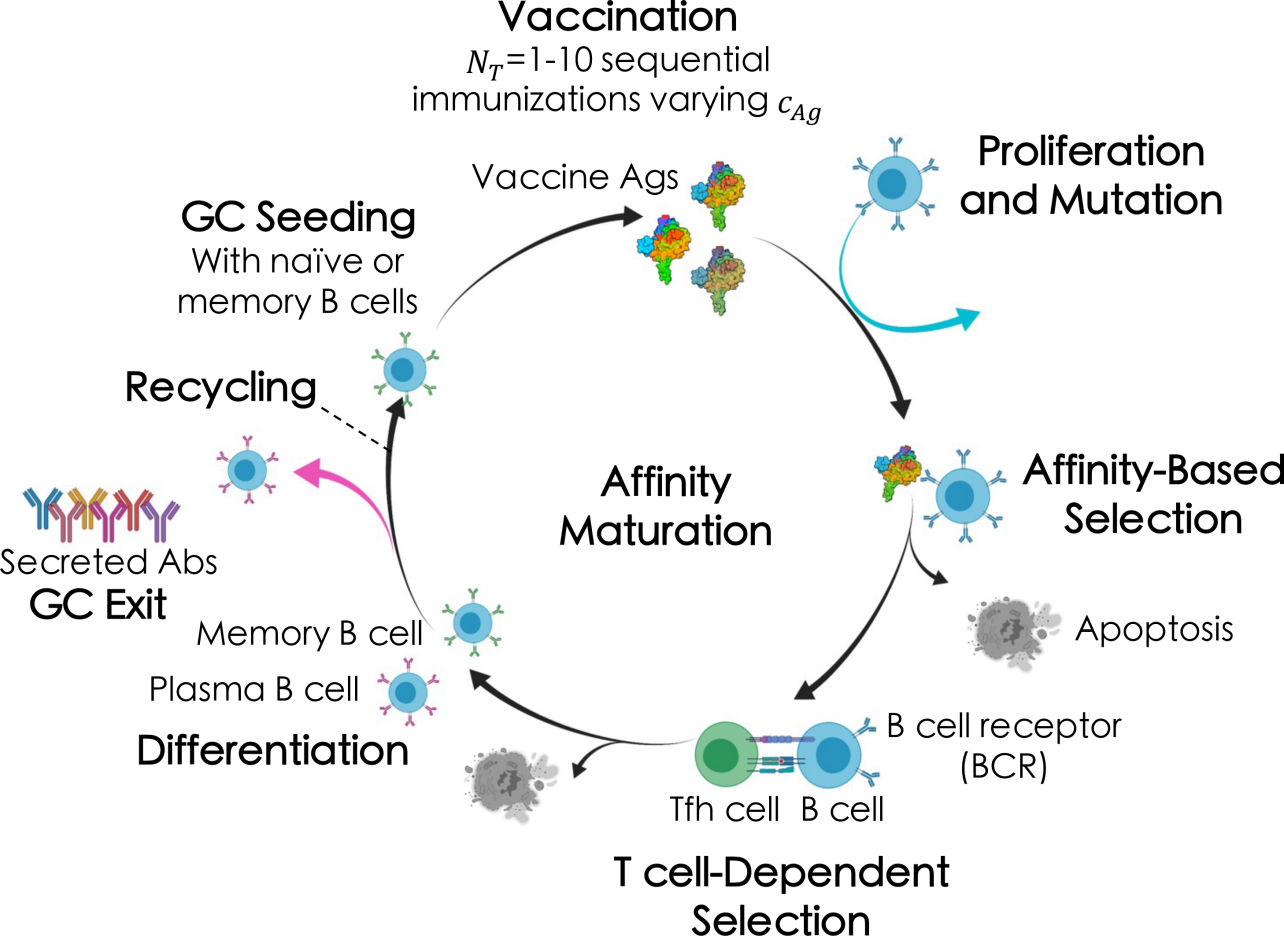
Broad overview of the affinity maturation (AM) process by which antibodies (Abs) evolve against vaccine-candidate antigens (Ags) in a germinal center (GC) reaction (see text for details). Here, we administered between one and ten total immunizations, *N*_*T*_ , of a single Ag, varying only the Ag concentration, *c*_*Ag*_ , in each immunization (indicated by the different shades of the Ags).

Briefly (see ensuing sections for more details), the AM process takes place within sites in lymph nodes called Germinal Centers, or GCs (**Figure 2**) (51, 58). After vaccination or natural infection, B cells whose receptors (BCRs) can weakly bind to the encountered antigen above some threshold affinity will seed a GC and start the process of AM. Inside the GC, the B cells replicate to increase the initial B cell population, after which they accumulate mutations in their surface receptor proteins. The B cells are then continuously recycled and selected based on their affinity for the antigen. After AM terminates, surviving B cells secrete their receptors as antibodies. In response to a single antigen administered at a fixed concentration, AM has been shown, through both computational and experimental studies, to produce mostly “strain-specific” antibodies (51, 59–62) These antibodies are generally ineffective against mutated antigens that may be encountered in the future. In contrast, administering sequential vaccine immunizations comprised of multiple different antigens or differing antigen concentrations serves to focus antibody responses on the conserved antigen regions, thus promoting the evolution of bnAbs (11, 13, 24, 63).

The AM model used in this work is the same as that developed and employed by us in our recent past work (13), apart from a few key differences. First, we made a minor change to how GCs are seeded, wherein memory B cells are chosen based on their prevalence in the memory B cell population (i.e., at the B cell level, rather than at the clonal level, as before). A second minor change we made was to let some B cells from the final cycle of AM differentiate into, and be added to the pool of, memory B cells that could seed future GCs (vs. all B cells differentiating into plasma B cells at the end of AM, as before). In the following sections, we provide only a broad overview of the key steps involved in the AM aspects of the model, focusing instead on the coupling to a deep reinforcement learning (DRL) algorithm. We refer readers to our past work (13) for more details on the model and its parameters.

#### Sequence modeling and binding free energy calculations

Sequences of the B cell receptor paratope (which we will simply refer to as BCR) and antigen epitope (which we will simply refer to as the antigen) are represented by strings of residues of equal length (total: 46 residues; 28 variable; 18 conserved). Antigen residues can take on only discrete values of +1 for conserved and native variable residues, or values of -1 for mutated variable residues, which is set at the beginning of the simulation and does not change throughout. BCR residue values are instead pulled from a continuous and bounded uniform distribution (see past work (13) for details) that is shifted towards higher values for conserved residues, reflecting the selection pressures imposed during the germline-targeting scheme (64–66) assumed to take place beforehand (see next section). BCRs are allowed to evolve in response to the administered antigen(s), as subsequently described.

Binding free energies are represented by a pairwise summation of the interactions between contacting BCR and antigen residues, which are in turn calculated as the product of their individual residue values (Eqn. 1):


(1) $$E=\sum_{all\;residues\;k}BCR(k)\cdot antigen(k)$$

In this case, more positive energy (*E* ) values correspond to higher binding free energies, the units of which are expressed in thermal energy (*k*_*B*_ *T* ). It is further assumed that a binding free energy of 9 *k*_*B*_ *T* is required for a B cell to seed a germinal center (GC) (13).

#### Seeding of germinal centers

As in our past work (13), we assume a germline-targeting immunogen has been administered prior to vaccination to activate the desired bnAb precursor B cells (22, 64–67). As a result, the initial sequences of our seeding BCRs include residues that are slightly biased towards positive values if they directly contact conserved antigenic residues. Following the presumed germline-targeting scheme and upon administration of the first immunization step in the vaccination protocol, we assume that each GC is seeded by 10 B cells (62).

#### Expansion and somatic hypermutation

Upon seeding a GC, the 10 B cells proliferate, without mutation, in what is known as the “dark zone” for around nine days (2^9), reaching a starting population size of 5,120 B cells in each GC. The B cells then begin to undergo somatic hypermutation, during which mutations are acquired in their B cell receptors (BCRs) at a rate of 0.14 mutations per sequence per division (61), with each B cell dividing twice per GC cycle (68). For those B cells that acquire a mutation in a given round of AM, experiments then suggest that somatic hypermutation causes lethal mutations in ~50% of the cells, silent mutations in ~30% of the cells, and affects the BCR/antigen binding free energy ~20% of the time (61). At present, our model does not consider the effects of BCR framework mutations.

Furthermore, energy-affecting mutations—which are chosen to occur randomly throughout the BCR sequence—have been shown to improve the binding free energy just 5-10% of the time for protein-protein binding events (69). This fact is replicated here by sampling changes in the BCR/antigen binding free energy from an empirically-derived, bounded lognormal distribution (see past work (13) for further details). Additionally, as in our past work (13), we account for the steric and conformational effects on BCR/antigen interactions of antigenic mutations that introduce loops to physically shield BCRs from binding to the conserved residues (70). We do this through the inclusion of a parameter that scales down (weakens) a BCR’s interactions with a randomly-chosen conserved antigenic residue if the BCR evolves a mutation that *strengthens* interactions with a variable antigenic residue, and vice versa.

#### B cell selection

Following somatic hypermutation, B cells compete with one another in what is known as the “light zone” for binding to the antigen, and upon binding, for receiving survival/proliferation signals from T helper cells (71). BCRs which bind more strongly to the antigen are able to internalize, break down, and display larger amounts of antigen on their surface, which in turn increases their chance of receiving survival/proliferation signals from T helper cells (71). B cells that do not receive these signals undergo apoptosis (72–74). The behavior outlined above is implemented *via* Eqn. 2:


(2) $$P_{internalize}=\frac{c_{i}\cdot e^{e_{scale}(E_{i}^{j}-E_{act})}}{1+c_{i}\cdot e^{e_{scale}(E_{i}^{j}-E_{act})}}$$

where the probability of a B cell *j* internalizing antigen *i* is assumed to depend on the concentration of the antigen *c*_*i*_ , as well as the binding free energy $E_{i}^{j}$ , the activation energy *E*_*act*_ , and an energy scaling parameter *e*_*scale*_ that serves as a pseudo inverse temperature (chosen to be 0.08 k_B_T^-1^) (13). The B cells that survive this first step are then ranked by their antigen binding free energies, and the top 70% receive productive signals from T helper cells. These parameters were previously optimized by us to recapitulate experimental data upon immunization with a single antigen (51, 60–62), and they remain unchanged here.

#### B cell recycling and differentiation, and GC termination

The number of B cells that exit the GC after each round of AM is determined using a random binomial distribution, with an exit probability of 30%. The exiting cells are then added to the potential memory B cell pool that is used to seed new GCs upon subsequent immunizations. The other 70% of the surviving B cells are recycled for further rounds of mutation and selection. GCs terminate successfully when the B cell population recovers its initial size of 5,120 cells, or unsuccessfully if all of the B cells die.

#### Memory b cell selection for seeding subsequent GCs

For vaccination protocols consisting of more than one immunization, GCs formed upon the *nth* immunization are seeded with memory B cells produced during the previous immunizations. As in our past work (13), we assumed here that only memory B cells become activated upon subsequent immunizations, rather than or in addition to naïve B cells. Ten memory B cells were chosen to seed each new GC, with selection probabilities set equal to their relative prevalence in the memory B cell population.

#### Breadth calculations

To determine the breadth of the antibodies that result from each simulated vaccination protocol, we compute the binding free energy of each antibody against an artificial panel of 100 different antigens. The panel sequences contain the same conserved residue values (all +1) as the antigen(s) administered in the vaccine protocol, whereas the variable residues in the panel sequences have equal probability of taking on a +1 or -1 value and thus differ from the administered antigen sequence(s). Previously, we found that increasing the number of antigens on the panel to 1,000 had little-to-no effect on the resulting breadth values (13). After calculating the set of 100 binding free energies for a given antibody, the breadth of the antibody is computed as the fraction of panel antigens for which the antibody binds above a threshold value (*E*_*th*_ ) of 12 *k*_*B*_ *T* . This value was chosen so that few antibodies produced from a single immunization are deemed “broad”, but so that antibodies *can* achieve high breadth in some cases after multiple immunizations, in line with experiments. To calculate meaningful statistics, each simulated vaccination protocol is performed 100 times, representing 100 GC reactions, and then performed in triplicate. This allows us to calculate the mean clonal breadth for each vaccination protocol (Eqn. 3):


(3) $$mean\;breadth=\frac{1}{N_{clones\;in\;all\;GCs}}\sum_{all\;clones\;j\;in\;all\;GCs}\frac{1}{N_{panel\;Ags}}\sum_{all\;panel\;Ags\;i}E_{ij}>E_{th}\;$$

We can then calculate the mean bnAb titers produced per GC as shown in Eqn. 4 below, using a value of 0.8 as the threshold for defining a bnAb as in our past work:


(4) $$bNAb\;titers/GC=\frac{1}{N_{GCs}\;}\sum_{all\;clones\;j\;in\;all\;GCs}breadth(j)>0.8\;$$

#### Frustration

Exposing the immune system to multiple diverse antigens results in conflicting selection forces in the AM process. The degree to which these selection forces throw the evolving B cell population off of steady state has been termed *frustration* in the past (11, 12). Varying the antigen sequence and/or concentration (among other potential variables) frustrates the evolutionary process, and in response the B cell population is focused towards strengthening binding to conserved antigenic sites in order to survive. Imposing too much frustration during vaccination results in the survival of only a few very good (high-breadth) B cells, while imposing too little frustration results in the survival of primarily low-breadth (or “strain-specific”) B cells. Indeed, previous works have shown that there is an optimal level of frustration to impose in each immunization which maximizes both the quality and quantity/titers of high-breadth antibodies (e.g., bnAbs) (13, 24). Here, the frustration, *F*_*i*_ , of a single immunization is represented by a linear combination of the antigen mutational distance and inverse antigen concentration (Eqn. 5), as determined in our past work (13):


(5) $$F_{i}=d_{i}+w_{i}\cdot \frac{1}{c_{i}}$$

Where *d_i_* is the mutational distance, *c_i_* is the concentration (as in Eqn. 2), and *w_i_* is an empirical weighting factor for the two components [here, we used a value of 24.6, as in our past work (13)]. In our simulations, *d_i_* was kept constant, and so frustration was modulated only through changing the concentration of the administered antigen in each immunization.

### Coupling the AM model with deep reinforcement learning

#### Background and nomenclature

Reinforcement learning (RL) is a subfield within machine learning that seeks to find near-optimal solutions to complex, stochastic problems that are intractable to solve analytically. For a detailed description of the application of RL in both biological and artificial systems, readers are directed to the comprehensive text by Sutton and Barto (75), and to the free online resource Spinning Up by OpenAI (76) for an introduction to both RL and DRL. DRL expands upon standard RL by employing neural networks to represent the mapping between states and actions. Below, we provide a high-level overview of DRL, then describe how DRL is coupled to our model of affinity maturation.

Typically, agent-based modeling simulations progress without any outside intervention. That is, the parameters which govern the environment are set at the onset of the simulation and do not change once the simulation has commenced. When coupling DRL to agent-based models, *actions* are chosen by the DRL agent during the simulation which drive the parameters toward optimal, or near-optimal, values, based on maximizing a user-defined *reward* function. The mapping from *states* of the environment leads to actions (or a distribution over actions) constitutes a *policy*, or strategy, which is then optimized to maximize the chosen cumulative reward.

A DRL algorithm contains a few pieces: first, an environment, which can best be described as a Markov decision process with several core components, namely, a state space *S* , an action space *A* , a reward function *r*, and transition dynamics (i.e., the simulation environment, *ℰ* ); second, it contains a policy or agent, *π* , which maps states to a distribution of actions. It should be noted the DRL environment and DRL agent are separate entities from the *simulation* environment and agents within the agent-based model itself. A *state*, *s*∈*S* , is the complete description of all values within the agent-based model at each step. As the simulation proceeds, the agent-based model will maintain an internal state, and the DRL agent will make observations *o*=*O*(*s*) which may limit the amount of information available (see *Observation Space* below). An *action*, *a*∈*A*, is an input parameter selected by the DRL agent which then feeds into the agent-based model at a given time step *t*. The *simulation environment*, *ℰ* , is represented as a black box: the DRL agent takes an action *a*_*t*_ at time *t* , given state *s*_*t*_, and then updates its observation to *s*_*t*+1_ . The trajectory taken during a simulation is then referred to as an *episode*.

The actions a DRL agent takes *π* : *S* ↦ *P*(*A*). The policy maps a given state to a probability distribution of possible actions. Here, we use the Proximal Policy Optimization (PPO) reinforcement learning algorithm (77). Implementation of the PPO algorithm was obtained from OpenAI Baselines and utilized the open-source machine learning platform and numerical computational library TensorFlow. Open AI’s Gym interface was used to create custom DRL agents by serving as a standardized platform to connect the custom-built AM environment with the PPO algorithm. For a rigorous discussion of the PPO algorithm, we direct readers to the original publication (77).

Finally, the reward function, *r* :*S*×*A*×*S*↦*ℝ* , maps the state at a given time (*s*_*t*_) , action (*a*_*t*_) , and the subsequent state (*s*_*t*+1_) to a scalar value *ℝ* . The DRL agent then receives the *return*, which is the cumulative future reward from time *t* to the end of the episode, discounted by a *discount factor*, *γ*∈(0, 1] . The discount factor determines the relative weight of immediate versus future rewards. The return is thus calculated *via* Eqn. 6 below.


(6) $$R_{t}=\sum_{t=t^{\prime }}^{t_{f}}r_{t^{\prime }}\gamma^{t^{\prime }-t}$$

Here, *R*_*t*_ is the return, *r*_*t* ^′^_ is the initial reward at time *t* , *t*_*f*_ is the terminal time, and *γ* ^*t* ^′^−*t*^ is the discount factor. The workflow of DRL is then as follows: The DRL agent makes an observation, *s*_*t*_, then using the current policy, *π* , takes an action, *a*_*t*_ , which results in a new state, *s*_*t*+1_ , and subsequent reward, *r*_*t*_  . These interactions can be represented as a feedback loop (**Figure 1**).

#### Affinity maturation as a DRL environment

In the present work, an episode is defined as 100 vaccination protocols (or GC reactions) consisting of one or more immunizations, or the time until the B cell population dies, and the GC reaction terminates unsuccessfully. A DRL step is defined as a single immunization and finishes when the B cell population recovers its initial size of 5,120 cells. The simulations were all run for 48 hours of wall-time and typically consisted of 100,000 - 150,000 DRL steps.

##### Observation space

Observations were designed to reflect the components of the immune system that would be activated upon/respond to a subsequent immunization—i.e., the memory B cell population produced in the prior immunization—and relevant metrics that can be observed clinically after each immunization, namely variables characterizing the distribution of breadth of the total memory B cell population produced after the 100 GC reactions. To this end, our observations include: (1) the number of bnAb-producing memory B cells, *N*_*bnAbs*_ , and (2) non-bnAb producing memory B cells, *N*_*non* −*bnAbs*_ , divided by a scalar (the initial B cell population size of 5,120×*N*_*GCs*_ ); (3) the weighted mean breadth ( $\bar{ℬ}=(N_{B,i}{ℬ}_{i})/\sum N_{B,i}$ ), where *ℬ*_*i*_ is the breadth of clone *i* and *N*_*B*,*i*_ is the number of B cells in clone *i* ; and (4), the weighted standard deviation of breadth ( $\sigma_{ℬ}=\sqrt{\sum {\left(N_{B,i}{ℬ}_{i}\right)}^{2}/\sum N_{B,i}}\;$ ).

##### Action space

The actions taken by the DRL agent correspond to the level of frustration imposed during each immunization on B cell receptor evolution (see earlier section on *Frustration* for more details). For immunization *i* , an individual action, *a*_*t*_ , is selected and then scaled and shifted according to Eqn. 7, essentially converting the action into a frustration value. While the action space is bounded to the range of *A*∈[−100, 100], in practice most of the selected actions fall in the range of [−1, 1] , resulting in actions that typically fall within the range of [20, 40] after being scaled and shifted. In our past work, this was found to be the relevant range of frustration values to explore in a single immunization.


(7) $$F_{i}=10\times a_{t}+30$$

*F*_*i*_ is then converted to an antigen concentration value using Eqn. 5, for later use in Eqn. 2.

##### Reward function

In addition to observing the state of the system after each immunization/step, the DRL agent receives a reward following each immunization. To explore the effects of optimizing for different metrics of protection upon vaccination on the resulting DRL policy (or optimal temporal frustration profile), we tested two relevant reward functions. First, we set our reward function to be the number of bnAb-producing plasma B cells, or essentially the produced bnAb titers, scaled by the initial B cell population size of 5,120 cells. We note that while the observation space is based on *memory* B cells, the reward function is based on *plasma* B cells, since these cells are responsible for secreting the antibodies that will seek out and fight the infection and thus directly reflect the quality of the immune response. In a second set of experiments, we set our reward function to be the fraction of bnAb-producing plasma B cells (or simply bnAbs produced) out of the total number of nAbs produced.

##### Hyperparameters and hardware

The following hyperparameters were used and are defined using the terminology seen in PPO baselines from OpenAI. Unless explicitly stated, the hyperparameters were kept at their default values. The DRL agent performed 32 steps before then performing stochastic gradient descent updates on four minibatches for four epochs. These parameters set the rate at which the policy is updated. To guide the step size of the policy update, a clipping range of 0.2 was used, with a value of 0.5 for the maximum value of gradient clipping. Evaluations were conducted every 5000 episodes, using 100 episodes per evaluation. In our preliminary studies using this system, the DRL agent adequately explored a wide range of frustration values, and thus the entropy coefficient for this study was set to zero. To shape the reward function, and to balance the variance-vs-bias of the Generalized Advantage Estimator (78), we selected gamma and lambda to be 0.99 and 0.95, respectively. The value function loss coefficient was set to 0.5. All simulations were performed on the Alpine supercomputing cluster at the University of Colorado, Boulder on 64-core AMD Milan CPUs.

## Results

### Overview and justification of approach

Previous work using this AM model (13) (without DRL) varied the frustration imposed on GC reactions across two immunizations and revealed an optimum level of imposed frustration exists for each immunization to maximize the number of bnAbs produced. Further, the optimum level of frustration was found to increase from the first immunization to the second. Here, rather than manually adjusting the level of imposed frustration to determine the optimum temporal frustration profile, a DRL agent was responsible for selecting frustration values and learning to map these values to the resulting immune response for a given number of immunizations. In this manner, DRL efficiently steered the choice of frustration values towards those values that directly affect the breadth of binding (or other properties of interest) of the evolving antibody population, thereby speeding up exploration of the *relevant* regions of the frustration landscape.

**Figure 3:**
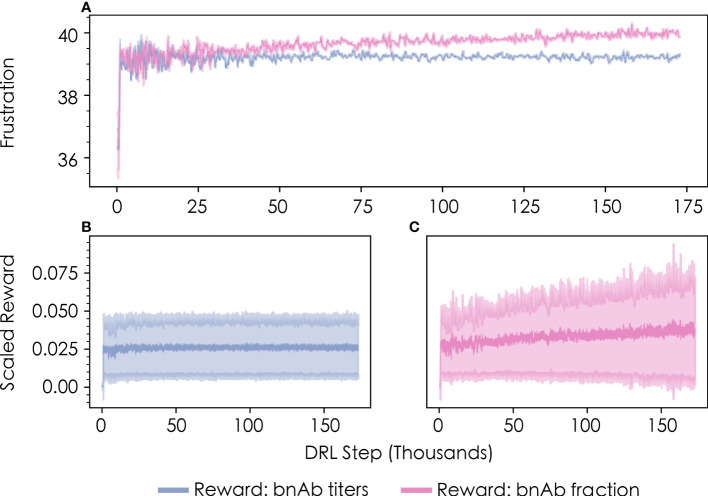
Convergence profiles of **(A)** the level of frustration imposed on GC reactions in the first vaccine immunization, as chosen by the DRL agent, and **(B, C)** the corresponding scaled reward values obtained by the DRL agent. Results are shown in blue for the bnAb titers reward function, and in pink for the bnAb fraction reward function. The data shown represents a rolling average with 500 DRL steps for both the mean and standard deviation (shown in lighter colors).

Such sequence-property relationships are usually impossible to know *a priori*. In this case, however, previous work (13) with this model has shown that imposing frustration values below ~35 or above ~45 in the first vaccine immunization results in relatively low bnAb titers—the former due to the production of primarily low-breadth antibodies with few mutations, and the latter due to the production of very few antibodies overall. The DRL agent reached this same conclusion after the first vaccine immunization extremely quickly (within a few thousand DRL steps/immunizations), without needing to spend much time exploring these unimportant regions of the frustration landscape. This finding was observed to be true for both metrics of protection (**Figure 3A**, “bnAb titers”; **Figure 3B**, “bnAb fraction”).

Consistent with results from previous work (13), we also find that the level of frustration that maximizes the bnAb titers produced in the first vaccine immunization—as chosen by the DRL agent—is a value of *F*_1_≈39 (**Figure 3A**, blue line). The optimal frustration value for maximizing the fraction of bnAbs produced in the first vaccine immunization is observed to be slightly higher, at a value of *F*_1_≈40 (**Figure 3A**, pink line). We describe why these differences emerge in the following section, which provides an analysis of the temporal frustration profiles across multiple immunizations for the two reward functions. **Figures 3B**, **C** show the corresponding scaled reward values obtained by the DRL agent as a function of the number of DRL steps/immunizations for the bnAb titers and bnAb fraction reward functions, respectively. The reward profiles closely parallel the frustration profiles show in **Figure 3A** in terms of the number of DRL steps needed to converge to an optimal value, indicating that sampling the optimal frustration value results in the optimal reward being obtained by the DRL agent, as expected.

### Optimal temporal immunization protocols differ for the two metrics of protection

**Figure 4:**
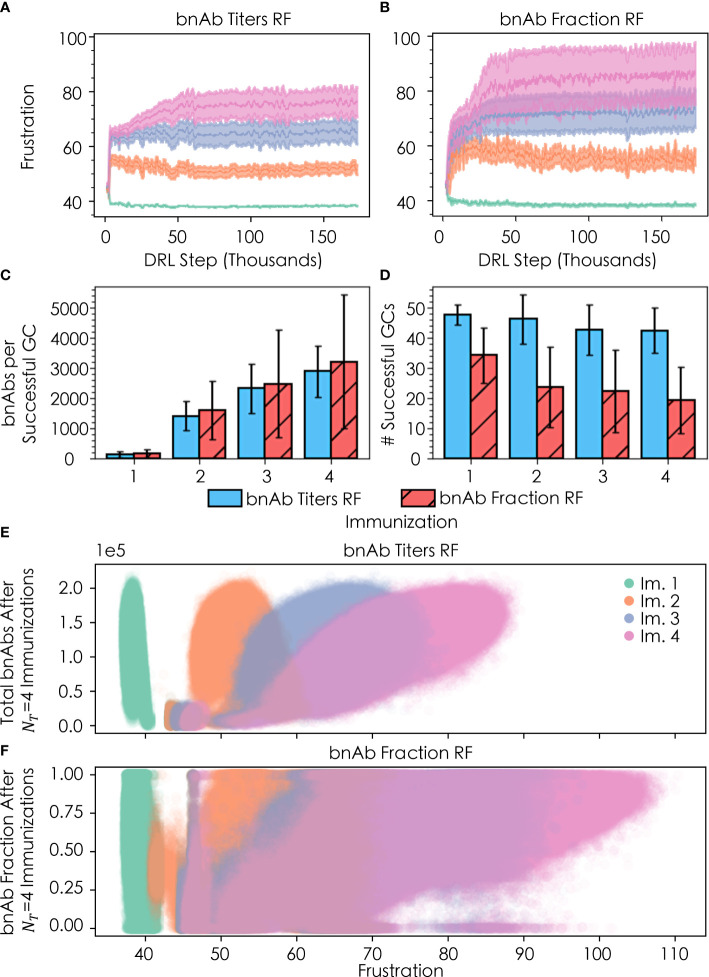
Convergence of frustration values *F*_1_ (green), *F*_2_ (orange), *F*_3_ (purple), and *F*_4_ (pink) for **(A)** the bnAb titers reward function (RF) and **(B)** the bnAb fraction RF, across four total immunizations; **(C)** bnAb titers produced per successful GC (out of n=100 GCs) for both RFs after each of the four immunizations; **(D)** number of successfully terminating GCs after each immunization for both RFs; **(E)** distribution of bnAb titer response for a given *F*_*i*_ after the four immunizations; and **(F)** fractional bnAb response for a given *F*_*i*_ after the four immunizations. In **(A, B)**, the rolling average +/- the rolling standard deviation is plotted using 500 DRL steps. In **(C, D)**, error bars are +/- the standard deviation of the respective metric (bnAb titers/successful GC and total successful GCs, respectively). In **(E, F)**, the y-values shown are the responses after administering all four immunizations; that is, the y-values are plotted as (*F*_*i*∈4_, *ℛ*_4_) for *ℛ*_4_≡ total bnAb titers and bnAb fraction, respectively.

We explored how changing the DRL reward function (or metric of protection) leads to changes in the optimal temporal frustration profile for a vaccination protocol with four sequential immunizations of a single antigen. Specifically, we performed simulations with the DRL reward function set to: (1) the total number of bnAbs produced (“bnAb titers”), enabling us to compare against our past work, and (2) the number of bnAbs produced divided by the total number of nAbs produced (“bnAb fraction”). We find that the optimal temporal frustration profile chosen by the DRL agent using the bnAb titers reward function reproduces the conclusion from our previous work that frustration should be optimally increased from the first to the second vaccine immunization in order to maximize bnAb titers (**Figure 4A**) (13). Furthermore, this fact is observed to hold true across all four immunizations, though, as discussed in the next section, the optimal difference in the level of frustration imposed on GC reactions between immunizations becomes increasingly small at high numbers of immunizations, *N*_*T*_ , in a given vaccine protocol. Similar to the results obtained with the bnAb titers reward function, using the bnAb fraction reward function we observe that the optimal frustration value increases across the four immunizations (**Figure 4B**).

Also in line with our past work (13), with the bnAb titers reward function we observe an increase in the mean number of bnAbs produced per successful GC (out of n=100 GCs) after each sequential immunization (**Figure 4C**), while the mean number of successful GCs decreases slightly with each subsequent immunization (**Figure 4D**). We note that while **Figures 4C**, **D** were constructed using all of the acquired data for each immunization, as discussed earlier, the DRL agent quickly arrives at the optimal frustration values, and thus the mean and standard deviation of the values in **Figures 4C**, **D** are highly representative of the optimal policy. The increases in bnAb production with each additional immunization may be attributed to the fact that the seeding B cells bind increasingly strongly to the conserved antigen residues with each ensuing immunization, so their paths towards becoming bnAbs are shorter and more streamlined. In addition, the increased binding strength to conserved antigen residues with subsequent immunizations provides B cells with an increased tolerance for higher frustration values that serves to further focus B cell responses on the conserved antigen residues that confer breadth and bnAb-like character to the B cell receptors. Similar to the results obtained with the bnAb titers reward function, results obtained with the bnAb fraction reward function show an increase in the number of bnAbs produced per successful GC with each additional immunization (**Figure 4C**). The use of the bnAb fraction reward function results in the production of slightly more bnAbs per successful GC, though this occurs at a notable cost to the number of GCs that survive after a given immunization (**Figure 4D**).

Differences emerge among the two reward functions when comparing the final bnAb titers (**Figure 4E**) and fraction of bnAbs (**Figure 4F**) that result after all four vaccine immunizations have been administered, as a function of the level of frustration chosen by the DRL agent for immunization. With the bnAb titers reward function, the DRL agent initially chooses many similar frustration values between 43 and 47 for all four immunizations (**Figure 4A**), corresponding to the small elliptical-shaped clusters in **Figure 4E** (note, the cluster for immunization 2 overlays that of immunization 1). With this temporal frustration profile (or policy), relatively low bnAb titers result, leading to low rewards for the DRL agent. Continuing its exploration of the frustration landscape, the DRL agent then shifts to testing a broader range of frustration values, converging on a lower frustration value for the first immunization (*F*_1_≈39 , as stated earlier) and higher frustration values in the subsequent immunizations (*F*_2_≈52, *F*_3_≈65, and *F*_4_≈76). The lower frustration in the first immunization serves to maintain a relatively high mean GC success rate (**Figure 4D**), leading to a large B cell population that can be translated into high bnAb titers (and high rewards) in response to the high levels of frustration imposed in the ensuing immunizations (**Figure 4E**). In our past work, manually exploring this landscape led us to conclude that a frustration value of *F*_2_≈45 was optimal in the second vaccine immunization (13). While this initially looked promising to the DRL agent as well, due to the changes in how memory B cells were chosen (see Methods), the agent quickly found that higher rewards could be achieved at *F*_2_≈52 . We found that when reverting to our previous model—but keeping the DRL agent in the driver’s seat—the agent converged on the same frustration value as we saw in our past work. This observation highlights a major benefit of using DRL to steer agent-based models of biological processes, which is that DRL is able to adapt quickly and efficiently to changes in agent-based models that are made to better reflect new biological insights and/or changing circumstances, as they become available.

With the bnAb fraction reward function, the DRL agent again begins by choosing many similar frustration values between 43 and 47 for all four immunizations (**Figure 4B**). However, in this case, at these frustration values the DRL agent can *occasionally* receive high rewards (bnAb fractions near 1.0) after the third and fourth immunizations. This is possible because the high frustration in the first vaccine immunization serves to immediately focus B cells on conserved antigenic sites, while also promoting an extremely high GC extinction rate (i.e., less than ~10 out of 100 GCs survive) to drive down the number of GCs that must primarily produce bnAbs in order to collectively achieve a high bnAb fraction. By the third and fourth immunizations (i.e., with sufficient maturation time), the few surviving GCs do occasionally all produce high fractions of bnAbs and thus stochastically produce high rewards, while the produced bnAb titers remains low. Following the sampling of this first policy (temporal frustration profile), the DRL agent then shifts to explore a higher and broader range of frustration values to identify a policy that will achieve more consistent rewards. Similar to the case with the bnAb titers reward function, the DRL agent converges on a lower frustration value for the first immunization (*F*_1_≈40 , as stated earlier) and higher frustration values in the subsequent immunizations (*F*_2_≈55, *F*_3_≈73, and *F*_4_≈86). Once again, the lower frustration in the first immunization serves to increase the mean GC success rate (**Figure 4D**), maintaining a larger pool of B cells that can be evolved into bnAbs in response to the high levels of frustration imposed in the subsequent immunizations (**Figure 4F**). Here, however, the frustration values are much higher in these later immunizations than with the bnAb titers reward function in order to simultaneously eliminate non-bnAb-producing GCs and maximize the overall bnAb fraction. This is evidenced by the lower mean numbers of GCs that succeed after the second, third, and fourth immunizations with the bnAb fraction reward function (**Figure 4D**), yet this new policy still produces appreciable bnAb titers as well as a high bnAb fraction.

While the second policy discussed above with the bnAb fraction reward function leads to more consistent rewards for the DRL agent, the agent’s ability to arrive at this conclusion is complicated by the fact that both policies *can* produce similarly successful outcomes. This behavior is demonstrated in **Figure 4B** by the period of stagnation in the frustration values sampled by the DRL agent during the first ~25,000 DRL steps before the frustration jumps up, which occurs in an almost stepwise manner in the fourth and final immunization. Furthermore, this behavior explains the increased variance observed in the reward and frustration curves (**Figures 4A, B**, respectively) as well as in the range of frustration values explored (**Figures 4E, F**) with the bnAb fraction reward function compared to the bnAb titers reward function. Specifically, we can see in both the variance of **Figure 4B** and spread of **Figure 4F** that the DRL agent can receive reward values for bnAb fractions between 0 and 1.0 for *F*_4_∈[60, 70] , though, in this frustration range, higher bnAb fractions are observed—and thus rewards are received—only sporadically. As a result, the agent must continue to raise *F*_4_ until it arrives at a frustration regime with a more persistent reward, precisely, within *F*_4_∈(70, 105] . The ability of DRL to navigate through this jagged reward space and arrive at an optimal policy further exemplifies its usefulness as a tool in biological simulations.

### A sustained BnAb response is revealed at high numbers of minimally-tailored immunizations

Here, we sought to take advantage of the speed with which DRL can navigate multidimensional landscapes by exploring a large number of immunizations (*N*_*T*_=10) to understand if we eventually reach a point at which additional immunizations lead to diminishing returns in the titers or fraction of bnAbs produced. If so, such results could offer practical considerations for manufacturing vaccines against highly mutable pathogens; as we have seen with the COVID-19 pandemic, vaccine/pandemic fatigue (79) can occur even with a disease that can cause severe health effects. While a protocol with ten immunizations is likely unrealistic in most scenarios, such a deep exploration of this facet of the vaccine design space may provide new insights into AM dynamics and possible tradeoffs in antibody-mediated protection and vaccine manufacturability.

**Figure 5:**
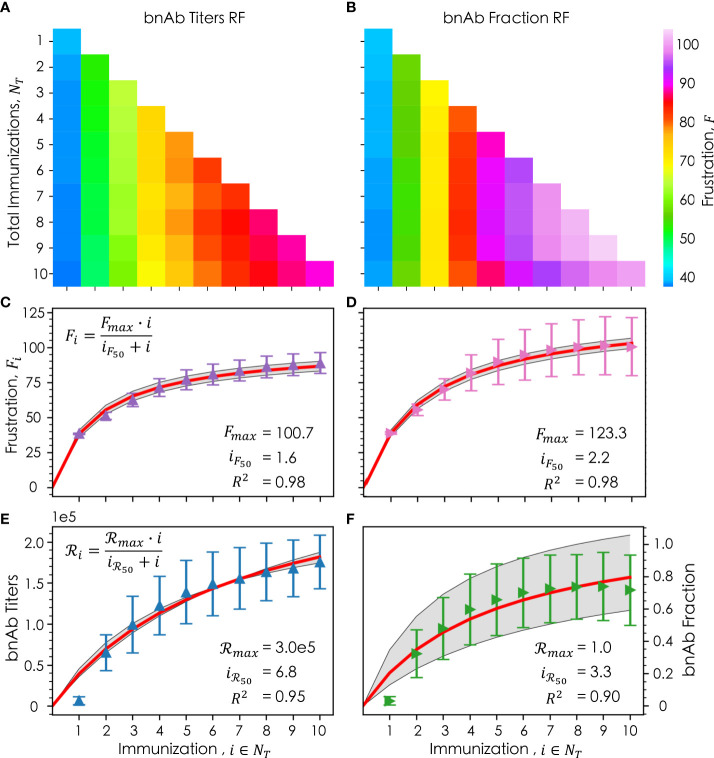
Frustration values of a given immunization, *i* , out of a total number of immunizations, *N*_*T*_ , where the column corresponds to the *i* ^*th*^ immunization, and the row corresponds to the total immunizations for both the **(A)** bnAb titers reward function and **(B)** bnAb fraction reward function; the average frustration for a given immunization, *F*_*i*_ , averaged across all *N*_*T*_ for **(C)** the bnAb titers reward function (purple) and **(D)** the bnAb fraction reward function (pink), with their respective Michaelis-Menten saturation fits (red) +/- the standard deviation (gray); the average reward for a given immunization, *ℛ*_*i*_ , for the bnAb titers reward function (**E**, blue) and bnAb fraction reward function (**F**, green), with their respective fits (red) and standard deviation of the fit (gray). In **(C–F)**, the error bars represent the standard deviation. Fitted parameters for the Michaelis-Menten model are shown in **(C–F)**.

We first sought to determine if and how an individual immunization’s frustration value, *F*_*i*_, changes as the total number of immunizations, *N*_*T*_, is increased. For the bnAb titers reward function, as *N*_*T*_ increases, *F*_*i*∈*N*_*T*__ tends to decrease (moving down each column in **Figure 5A**), the implications of which are discussed in detail in the following section. Interestingly, this trend does not appear to hold true for the bnAb fraction reward function (**Figure 5B**), except for at higher values of *i* (*i*= 7, 8, and 9). Instead, we observe that as *N*_*T*_ increases, *F*_*i*_ goes through a maximum for intermediate values of *i* (*i*= 4, 5, and 6). We also observe that for a given *N*_*T*_ , higher frustration values are required in each immunization in order to maximize the bnAb fraction versus the bnAb titers that are produced, consistent with our earlier results.

**Figures 5C** (bnAb titers reward function) and 5D (bnAb fraction reward function) show, as a function of *i*, the mean optimal *F*_*i*_ values after being averaged across all data at the relevant *N*_*T*_ (e.g., for *i*= 3, the data is averaged across all values of *F*_3_ in *N*_*T*_= 1, 2, and 3). The results show that for both reward functions, the differences in the mean optimal *F*_*i*_ value from one immunization to the next decrease as *i* increases, leading to a leveling out of the optimal frustration values. To understand this behavior more quantitatively, we fit our frustration data to a Michaelis-Menten saturation equation (Eqn. 8), leading to high *R* ^2^ values of 0.98 for both reward functions.


(8) $$F_{i}=\frac{F_{max}\cdot i}{i_{F_{50}}+i}$$

In this equation, *F*_*max*_ represents the maximum optimal level of frustration that can be imposed on GC reactions for a given reward function, with *F*_*Max*_ found to be 100.7 and 123.3 for the bnAb titers and bnAb fraction reward functions, respectively. The *i*_*F*_50__ parameter represents the number of immunizations that must be administered (at *their* optimal values of *F*_*i*_ ) in order to impose a level of frustration that is half that of *F*_*max*_ , with *i*_*F*_50__ found to be 1.6 and 2.2 for the bnAb titers and bnAb fraction reward functions, respectively. More intuitively, *i*_*F*_50__ can be thought of as the number of immunizations in which 50% of the diversity in the overall vaccine formulation is encompassed (e.g., in terms of how the antigen sequences or concentration profiles are designed). As such, our results suggest that approximately the first 3-4 immunizations for the bnAb titers reward function or the first 4-5 immunizations for the bnAb fraction reward function must be carefully tailored to impose the optimal frustration values—and in turn achieve the optimal reward values—in each immunization. Beyond these numbers of immunizations, our results suggest that immunizations can be more similarly designed or perhaps even kept the same to maintain a strong protective antibody response.

This latter point is exemplified in **Figures 5E, F**, which show as a function of *i* the mean achieved reward values *ℛ*_*i*_ averaged across all data at the relevant *N*_*T*_ , for the bnAb titers and bnAb fraction reward functions, respectively. The data has once again been fit to a Michaelis-Menten saturation curve (Eqn. 9), resulting in *R* ^2^ values of 0.95 and 0.90, respectively.


(9) $${ℛ}_{i}=\frac{{ℛ}_{max}\cdot i}{i_{{ℛ}_{50}}+i}$$

In this equation, *ℛ*_*max*_ represents the maximum reward that can be achieved, found to be a bnAb titer of 3.0x10^5^ (**Figure 5E**) and a bnAb fraction of 1.0 (**Figure 5F**). The *i*_*ℛ*_50__ parameter represents the number of immunizations that must be administered in order to achieve 50% of *ℛ*_*max*_, found to be 6.8 and 3.3 for the bnAb titers and bnAb fraction reward functions, respectively. Notably, the *i*_*ℛ*_50__ values are higher than the *i*_*F*_50__ values for both reward functions, though the difference is much greater for the bnAb titers reward function. This finding, discussed in more detail in the following section, is exciting, as it implies that even if the same or very similarly formulated immunizations are administered in later immunizations, the produced bnAb titers will continue to increase (at least up to *i*  =10). Though, we do observe the bnAb titers curve begin to level out as well at high values of *i* (**Figure 5E**). For the bnAb fraction reward value, this saturation behavior is much more pronounced by *i*  = 10, in line with the fact that the *i*_*ℛ*_50__ and *i*_*F*_50__ values are more similar.

## Discussion

The need for robust and easily accessible vaccines to aid in combating infectious diseases has been made evident by the recent COVID-19 pandemic, with a major issue arising in the lack of vaccine equity globally (80). Vaccine equity operates on the premise that regardless of geographical or socioeconomic status, all individuals worldwide should have access to vital vaccines. Universal vaccines—the colloquial “silver bullets”—could help to make this objective feasible. A universal vaccine aims to elicit broadly neutralizing antibodies (bnAbs), which target conserved sites on viral surface proteins and which therefore should be able to provide protection to an individual even if the virus mutates. However, having equal access to such a vaccine does not ensure all subpopulations will equally adhere to the vaccine’s immunization protocol, especially as more immunizations are required, or that all subpopulations will respond equally as effectively to the vaccine due to various sources of heterogeneity in their immune responses.

To account for these factors, one possible solution is to develop a set of minimally-tailored universal vaccines against a given hm-IDP. The feasibility of such an idea relies on how much the vaccination protocol would need to be altered to be sufficiently comprehensive (i.e., how large would the minimal set of universal vaccines need to be), which is currently unknown. To this end, we used a novel *in silico* approach combining a coarse-grained agent-based model of affinity maturation with deep reinforcement learning (DRL), to elucidate optimal temporal immunization protocols against hm-IDPs like HIV and influenza, for which universal vaccines remain elusive. Harnessing the power of DRL to increase the sampling efficiency of the relevant vaccine design space, we explored how the optimal temporal immunization protocol differs for different metrics of protection (DRL reward functions), namely the titers or fraction of bnAbs produced. We show that for both metrics of protection, a minimal set of bnAb-eliciting (and thus universal) vaccines may suffice to confer protection against a given hm-IDP. Specifically, our results suggest a set of 3-5 immunizations is sufficient to achieve a robust protective immune response. Additional immunizations may boost the immune response—increasing the bnAb titers—but do not require changes in formulation to elicit these titers.

High neutralizing antibody (nAb) titers are often the desired end goal for protecting against infectious diseases (bnAbs are the *ideal* target but have yet to be robustly and consistently elicited *via* vaccination). However, there are other factors that must be considered to ensure broad protection of the population against future pandemic events. One example could be the time-to-response, or the time it takes for the AM process to produce the first batch of bnAbs. Vaccines that aim to streamline the process of evolving bnAbs (i.e., evolve high antibody breadth *via* the fewest mutations possible), may enable the use of vaccines to curtail acute infectious disease outbreaks. Another example is that a reduction in sustained GC response and an increase in the *fraction* of bnAbs produced, rather than the total bnAb titers produced, could result in fewer off-target antibody responses. In the case of HIV, a recent study showed off-target antibodies elicited by an HIV vaccine actively hindered the acquisition of protection by destabilizing and degrading the components in the vaccine (81). Minimizing off-target responses by administering low-dose vaccines to operate at higher levels of *frustration* (see Methods) could also circumvent the theoretical concern that high-dose and/or adjuvanted vaccines may induce autoimmune responses or augment autoimmune disease activity (82).

For both metrics of protection we explored, our results indicate frustration should be optimally increased in each subsequent immunization, but that the degree to which the frustration should be increased from one immunization to the next differs for the different metrics of protection. More specifically, we find that the imposed frustration should be higher in each immunization to maximize the fraction of bnAbs produced rather than the total bnAb titers (for which the production of non-bnAb titers, or off-target antibody responses, may also be high). However, the use of the bnAb fraction reward function results in only about a 6.7% increase in the mean fraction of bnAbs produced compared with the bnAb titers reward function (**Figure 4C**). Additionally, this slight increase in the fraction of bnAbs produced comes at a steep reduction in the mean bnAb titers produced of nearly 50%, using the bnAb fraction versus bnAb titers reward function (**Figures 4C, D**). Thus, a vaccine strategy that aims to maximize the overall bnAb titers rather than the bnAb fraction may be more beneficial for the general populace, especially considering that more consistent results can be achieved with this approach (see earlier discussion of **Figures 4E, F**). For situations where it is crucial to minimize off-target responses, our results indicate there are multiple strategies for producing a high fraction of bnAbs. However, only one of these strategies is expected to elicit good results with any consistency, which entails ramping up the frustration in the later immunizations (here, starting in the fourth immunization), to ensure only a small handful of GCs survive that produce primarily bnAbs.

We also explored how the optimal temporal immunization protocols changed as the overall number of immunizations was increased between one and ten, leveraging DRL’s ability to efficiently parse such high-dimensional landscapes. Our results indicate vaccines must be tailored to be optimally different from one another (e.g., in terms of the antigen sequences or concentrations) in the first 3 to 5 immunizations in order to achieve broadly protective immune responses, based on either metric of protection explored, providing support for our idea of “minimally-tailored” universal vaccines. Beyond this number of immunizations, our results indicate that further immunizations need be designed to be only minimally different, if at all, from the prior immunizations, without causing a diminished immune response upon administration. As such, these later immunizations may be useful as boosters, akin to those administered for COVID-19, without reformulation.

In line with these observations, we found in our past work that for a total of two immunizations (*N*_*T*_=2), there is an optimal frustration to impose in the first immunization (*F*_1_) that enables maximum bnAb titers to be produced (if *F*_2_  is also at *its* optimal value), and that if *F*_1_ is decreased below its optimal value, this enables fewer bnAbs to be produced after the second immunization. This was found to be true regardless of how much *F*_2_ is increased to try and refocus B cell receptors on conserved antigen residues, as extensive B cell death occurred before a high enough frustration could be imposed to recoup the losses in the produced bnAb titers. Here, our results suggest that this effect can be mitigated by increasing *N*_*T*_, which will serve to slowly refocus B cell receptors on conserved sites over several sequential immunizations, thus minimizing B cell death along the way and producing high bnAb titers. In other words, our results indicate that the optimal level of frustration to impose in any given immunization depends on the total number of immunizations in the vaccine protocol. Alternatively, one can adopt the perspective that increasing *N*_*T*_ may serve as a remedy for administering sub-optimal levels of frustration in the previous immunization(s). However, such a remedy may run into practical considerations in terms of administration costs (manufacturing costs would presumably remain relatively low due to the high similarity of the later immunizations), or issues with maintaining high vaccination adherence rates in certain subpopulations and/or geographical locations.

The scope of our results is limited in that the model we employed uses coarse-grained representations for the antigen sequences, simplifying the physics and focusing our investigation on the population-level dynamics of the immune response. As previously mentioned, this work explores the immune dynamics of AM *after* a successful germline-targeting scheme has been presumed to have taken place to recruit the desired precursor B cells to germinal centers. Recent advances in targeting germline B cells known to evolve into VRC01-class, anti-HIV bnAbs have demonstrated some success (22, 64, 66, 83, 84), but success has yet to be realized in a clinical setting. The variance of germline precursor frequencies, as well as the effects of polyclonal Fc-mediated antibody responses also play important roles in vaccine design and warrant further investigation. Moreover, as previously mentioned, recent studies have demonstrated the importance of Ag capture in the AM process (52, 55), which we plan to incorporate into our model in the future. As the complexities of AM continue to be unraveled, so too shall our models evolve to better recapitulate the *in vitro* and *in vivo* findings of the field. Future studies with this DRL-driven computational framework will be performed to investigate if the results derived herein—which were based on modulating frustration only through changes in the temporal antigen concentration profiles—may change when allowing the DRL agent to vary the administered antigen sequences, and ultimately to vary both variables simultaneously. In addition, the capability of DRL to efficiently navigate complex landscapes will be further leveraged in future studies with models of AM that operate at the amino acid sequence level, such as a model recently developed by us (24), for which the design space is considerably larger. We look forward to future experimental studies that may enable validation of our computational findings that: (1) if an immunization is administered at a sub-optimal setting (e.g., at a setting that produces less bnAb titers than at a different setting), the production of high bnAb titers may be rescued by administering several additional immunizations designed to be highly similar to one another and to the prior immunization, and (2) that the imposed frustration should be slightly higher in each immunization to reduce off-target responses, versus simply maximizing the production of bnAbs.

## Data availability statement

The raw data supporting the conclusions of this article will be made available by the authors, without undue reservation.

## Author contributions

Author contributions: KS, BP, and JF designed research. KS, BP, JF, DO, and CW performed research. KS, BP, JF, and DO analyzed data, and KS, BP, and JF wrote the paper. All authors contributed to the article and approved the submitted version.

## Funding

This work utilized resources from the University of Colorado Boulder Research Computing Group, which is supported by the National Science Foundation (awards ACI-1532235 and ACI-1532236), the University of Colorado Boulder, and Colorado State University.

## Acknowledgments

This work was performed under the auspices of the U.S. Department of Energy by Lawrence Livermore National Laboratory under contract DE-AC52-07NA27344. Lawrence Livermore National Security, LLC. LLNL-JRNL-838776.

## Conflict of interest

The authors declare that the research was conducted in the absence of any commercial or financial relationships that could be construed as a potential conflict of interest.

## Publisher’s note

All claims expressed in this article are solely those of the authors and do not necessarily represent those of their affiliated organizations, or those of the publisher, the editors and the reviewers. Any product that may be evaluated in this article, or claim that may be made by its manufacturer, is not guaranteed or endorsed by the publisher.
